# Современные возможности компьютерной томографии с контрастным усилением в диагностике аденом надпочечников

**DOI:** 10.14341/probl13670

**Published:** 2026-03-07

**Authors:** Н. В. Тарбаева, А. Шевэ, А. В. Манаев, Д. Г. Бельцевич, Н. М. Платонова, Е. А. Трошина, Г. А. Мельниченко, Н. Г. Мокрышева

**Affiliations:** Национальный медицинский исследовательский центр эндокринологии им. академика И.И. ДедоваРоссия; Endocrinology Research CentreRussian Federation

**Keywords:** первичный гиперальдостеронизм, синдром Кушинга, компьютерная томография, КТ-характеристики, primary aldosteronism, Cushing’s syndrome, computed tomography, CT characteristics

## Abstract

**ОБОСНОВАНИЕ:**

ОБОСНОВАНИЕ. Инциденталомы надпочечников распространены с выявляемостью до 7% среди пациентов старше 70 лет, из них до 25% являются функционально активными, приводя к тяжелому развитию клинических проявлений. Сохраняются проблемы недостаточной диагностики и отсутствия персонализированного подхода к ведению таких пациентов. Для их преодоления предлагается применение анализа КТ-изображений для разработки критериев неинвазивной диагностики, что является путем к совершенствованию персонализированного ведения больных.

**ЦЕЛЬ:**

ЦЕЛЬ. Анализ наличия статистически значимых корреляций между клинико-лабораторными характеристиками и признаками изображений КТ с контрастным усилением (КУ) аденом надпочечников.

**МАТЕРИАЛЫ И МЕТОДЫ:**

МАТЕРИАЛЫ И МЕТОДЫ. В одноцентровом несравнительном одномоментном ретроспективном исследовании проанализированы предоперационные изображения четырехфазного КТ с КУ аденом надпочечников. Гормональное обследование включало определение уровня альдостерона, ренина, кортизола в ходе ночного подавляющего дексаметазонового теста (ПДТ1), адренокортикотропного гормона (АКТГ), свободного кортизола суточной мочи (СКМ), дополнительно исследовался уровень калия и креатинина. Клинические данные включали артериальную гипертензию, нарушение углеводного обмена, дислипидемию, гипокалиемию и нарушение функции почек. Проведен сравнительный и корреляционных анализ между клинико-лабораторными показателями и характеристиками КТ с КУ.

**РЕЗУЛЬТАТЫ:**

РЕЗУЛЬТАТЫ. В исследование включены 254 пациента. Гормональная активность выявлена у 226 (89,0%) пациентов; у 28 (11,0%) пациентов гормональной активности не выявлено. Гормонально-неактивные аденомы отличались большими размерами (43,0 мм [32,7; 51,2] против 29,0 мм [20; 36], p<0,001), более высокой нативной плотностью (41,0 HU [36,0; 47,2] против 25,0 HU [12,0; 37,0], p<0,001) и меньшим венозным накоплением контраста (141,6% против 283,7%, p=0,001). При первичном гиперальдостеронизме (ПГА) аденомы были значительно меньше по размеру (20,0 мм [16,8; 25,0]) и имели более низкую нативную плотность (14,0 HU [4,0; 24,0]) по сравнению с кортизол-продуцирующими опухолями (34,0 мм [30,0; 38,0], 35,5 HU [30,0; 44,0], p<0,001). Наличие кальцинатов чаще регистрировалось при гиперсекреции кортизола по сравнению с ПГА (p=0,011). Корреляционный анализ выявил отрицательные связи между размерами аденомы и уровнями альдостерона (r=–0,504, p<0,001), а также АКТГ (r=–0,419, p<0,001), и положительные связи с уровнями кортизола после ПДТ1 (r=0,500, p<0,001), ренина (r=0,454, p<0,001) и калия (r=0,458, p<0,001).

**ЗАКЛЮЧЕНИЕ:**

ЗАКЛЮЧЕНИЕ. КТ-характеристики аденом надпочечников различаются в зависимости от гормональной активности и типа секреции, клинических проявлений.

## Abstract (en)

**BACKGROUND:**

BACKGROUND: Adrenal incidentalomas are common, with a detection rate of up to 7% in patients over 70 years of age. Of these, up to 25% are functionally active, leading to the development of severe clinical manifestations. Challenges of insufficient diagnosis and a lack of a personalized approach to the management of such patients persist. To overcome these challenges, the use of CT image analysis is proposed to develop criteria for non-invasive diagnosis, which is a pathway towards improving personalized patient management.

**AIM:**

AIM: Analysis of statistically significant correlations between clinical-laboratory parameters and contrast-enhanced (CE) CT imaging features of adrenal adenomas.

**MATERIALS AND METHODS:**

MATERIALS AND METHODS: A single-center, non-comparative, cross-sectional retrospective study analyzed preoperative images from four-phase CECT of adrenal adenomas. Hormonal workup included the determination of aldosterone, renin, cortisol levels during an overnight dexamethasone suppression test (DST), adrenocorticotropic hormone (ACTH), 24-hour urinary free cortisol (UFC); potassium and creatinine levels were also assessed. Clinical data included arterial hypertension, impaired carbohydrate metabolism, dyslipidemia, hypokalemia, and renal dysfunction. A comparative and correlation analysis was performed between clinical-laboratory parameters and CECT characteristics.

**RESULTS:**

RESULTS: The study included 254 patients. Hormonal activity was detected in 226 (89.0%) patients; 28 (11.0%) patients had non-functioning adenomas. Hormonally inactive adenomas were characterized by larger size (43.0 mm [32.7; 51.2] vs. 29.0 mm [20; 36], p<0.001), higher native density (41.0 HU [36.0; 47.2] vs. 25.0 HU [12.0; 37.0], p<0.001), and lower venous phase contrast enhancement (141.6% vs. 283.7%, p=0.001). In primary aldosteronism (PA), adenomas were significantly smaller (20.0 mm [16.8; 25.0]) and had lower native density (14.0 HU [4.0; 24.0]) compared to cortisol-producing tumors (34.0 mm [30.0; 38.0], 35.5 HU [30.0; 44.0], p<0.001). The presence of calcifications was more frequently observed in cortisol hypersecretion compared to PA (p=0.011). Correlation analysis revealed negative associations between adenoma size and levels of aldosterone (r=–0.504, p<0.001) and ACTH (r=–0.419, p<0.001), and positive associations with post-DST cortisol levels (r=0.500, p<0.001), renin (r = 0.454, p<0.001), and potassium (r=0.458, p<0.001).

**CONCLUSION:**

CONCLUSION: CT characteristics of adrenal adenomas vary depending on hormonal activity, type of secretion, and clinical manifestations.

## Обоснование

За последние четыре десятилетия в связи с увеличивающейся распространенностью и общедоступностью топических методов диагностики (КТ, магнитно-резонансная томография (МРТ), ультразвуковое исследование (УЗИ)) выявляемость образований надпочечников резко выросла.

По данным сводной статистики аутопсий, распространенность случайно обнаруженных опухолей надпочечника составляет в среднем 6%. По данным КТ, «случайные» образования надпочечника выявляются примерно у 4% обследованных пациентов. У лиц младше 30 лет инциденталома встречается примерно у 0,2% обследованных, тогда как у пациентов старше 70 лет ее частота увеличивается до 7% [1][2].

Согласно действующим клиническим рекомендациям, на момент первичной диагностики все пациенты с инциденталомой надпочечника должны пройти тщательное клиническое, лабораторное и визуализирующее обследование, чтобы оценить, является ли опухоль злокачественной, и определить наличие избытка гормонов надпочечников.

Большая часть (75%) этих образований представлены аденомами надпочечников. Аденома коркового вещества надпочечника — это новообразование, происходящее из клеток коркового вещества надпочечника, не имеющее морфологических признаков злокачественности. Аденомы коркового вещества надпочечника характеризуются гетерогенностью патологических и клинических проявлений, а также имеют различные гистопатологические варианты. При патоморфологическом исследовании типичные аденомы надпочечников весят менее 50 г и достигают диаметра до 5 см. Это четко очерченные солитарные опухоли, обычно ярко-желтого цвета из-за содержания липидов. Наличие в образовании липофусцина объясняет их более темный цвет — коричневый или черный. При микроскопическом исследовании типичные аденомы состоят из пролиферирующих прозрачных и компактных клеток. Иногда присутствуют увеличенные ядра, но атипия и митотическая активность встречаются очень редко [3].

Аденомы надпочечника могут быть гормонально неактивными или синтезировать/секретировать стероидные гормоны с субклиническими или явными клиническими проявлениями. До 25% из них могут быть функционально активными: при избыточной секреции глюкокортикоидов возникает состояние, получившее название функционально автономной продукции кортизола (ФАПК) или синдром Кушинга (СК), при гиперпродукции минералокортикоидов — первичный гиперальдостеронизм (ПГА) или синдром Конна (в случае солитарной гормон-продуцирующей опухоли) соответственно. Реже аденомы секретируют половые гормоны или имеют смешанную секрецию [4][5].

Значительная часть гормонально-активных образований надпочечников встречается у работоспособной части населения и сопровождается развитием тяжелой артериальной гипертензии, метаболических нарушений, сопряженных с высоким риском развития тяжелых сердечно-сосудистых осложнений, инвалидизации и преждевременной смерти. В настоящее время остаются нерешенными проблемы должной диагностики и разработки персонализированного ведения больных.

Из-за своей широкой распространенности аденомы надпочечников могут иметь различные КТ-характеристики. Таким образом, важно идентифицировать типичные и атипичные признаки КТ-изображений аденом надпочечников. Анализ изображений КТ адренокортикальных аденом позволяет получать дополнительную информацию о структуре и характеристиках тканей, что способствует более точной дифференцировке опухолей и улучшению диагностики.

## Цель исследования

Анализ наличия статистически значимых корреляций между клинико-лабораторными характеристиками и признаками изображений КТ с контрастным усилением (КУ) аденом надпочечников.

## Материалы и методы

## Место и время проведения исследования

Место проведения. ФГБУ «НМИЦ эндокринологии им. академика И.И. Дедова» Минздрава России, референс-центр лучевых методов диагностики.

Время исследования. Ноябрь 2024 — август 2025 гг.

## Изучаемые популяции (одна или несколько)

Целевая популяция определялась критериями включения и исключения.

Критерии включения: клинический диагноз гормонально-активной опухоли надпочечника (ФАПК, манифестная форма СК, ГА) или гормонально-неактивной опухоли надпочечника с неопределенным или злокачественным КТ-фенотипом, выполнение хирургического лечения, подтверждение диагноза аденомы по результатам патоморфологического исследования операционного материала, наличие данных четырехфазной КТ (нативная, артериальная, венозная, отсроченная фазы).

Критерии исключения: наличие артефактов в области надпочечников на изображениях КТ

## Способ формирования выборки из изучаемой популяции (или нескольких выборок из нескольких изучаемых популяций)

Участники исследования подбирались в соответствии с критериями отбора до включения в исследование с формированием «удобной» для наблюдения исследователями выборки пациентов с образованиями надпочечников, зарегистрированных в медицинской информационной системе.

## Дизайн исследования

Одноцентровое несравнительное одномоментное ретроспективное исследование.

## Методы

Показанием к оперативному лечению в случае гормонально-активных форм являлись:

манифестная форма СК

функционально-автономная продукция кортизола (ФАПК)

односторонняя форма ПГА

## Компьютерная томография

Четырехфазную КТ с КУ проводили с применением томографов Optima CT660 — 123 случая и Revolution CT — 131 случай (GE Healthcare, США). Контрастное усиление выполнялось с использованием двухколбового автоматического инжектора Medrad Stellant (Bayer) со скоростью подачи 3,5–4 мл/с. Артериальная фаза выполнялась на 10-й секунде после срабатывания триггера болюса, установленного на нисходящей аорте на уровне диафрагмы (120 HU). Венозная фаза выполнялась через 30 секунд после активации триггера, отсроченная фаза — через 10–15 минут после введения контрастного вещества.

## Формирование массива данных для анализа

Лабораторное обследование

Для подтверждения/исключения гиперкортицизма (ФАПК, манифестный СК) определяли:

- уровень кортизола в ходе ночного подавляющего теста с 1 мг дексаметазона (ПДТ1). Исследование уровня кортизола выполнено на электрохемилюминесцентных анализаторах фирмы Roche (Cobas 6000 Modulee 601) стандартными наборами F. Hoffmann-La Roche Ltd). Согласно действующим клиническим рекомендациям, пороговое значение составило 50 нмоль/л;

- уровень свободного кортизола в суточной моче иммунохемилюминесцентным методом на аппарате Vitros Eci с предварительной экстракцией эфиром. Референсный интервал: 60–413 нмоль/сут;

- уровень утреннего АКТГ на электрохемилюминесцентных анализаторах фирмы Roche (Cobas 6000 Modulee601) наборами F. Hoffmann-La Roche Ltd. Референсный интервал: 7,2–66 пг/мл.

Для подтверждения/исключения ПГА

- уровень альдостерона определялся на электрохемилюминесцентных анализаторах фирмы Roche (Cobas 6000 Modulee 601) наборами фирмы F. Hoffmann-La Roche Ltd). Референсный интервал: 48,7–653,7 пмоль/л;

- уровень прямого ренина определялся методом хемилюминесцентного иммуноанализа (CLIA) с помощью анализатора фирмы LIASON («Diasorin», Италия). Референсный интервал: 2,8–39,9 нмоль/л;

- уровень калия исследовался на биохимическом анализаторе Architect 8000 (Abbott Diagnostics, Abbot park, IL, USA). Референсный интервал: 3,5–5,1;

- уровень креатинина, глюкозы, показателей липидного обмена (общего холестерина, липопротеинов низкой плотности, липопротеинов высокой плотности, триглицериды), анализировлись на биохимическом анализаторе Architect 8000 (Abbott Diagnostics, Abbot park, IL, USA) стандартными наборами фирмы.

Клинические данные

Для оценки тяжести течения сопутствующих заболеваний/осложнений СК и ПГА, всем пациентам проведена оценка наличия/отсутствия ожирения (подсчитан ИМТ), АГ нарушений углеводного и липидного обмена, нарушений функций почек.

Диагноз артериальной гипертензии (АГ) устанавливался при многократном повышении АД выше 140/90 мм рт.ст. При наличии у пациента нецелевых показателей глюкозы плазмы крови натощак (>6,1 ммоль/л), для исключения наличия нарушений углеводного обмена проводился пероральный глюкозотолерантный тест.

Дислипидемия устанавливалась на основании нарушения показателей липидного спектра (нецелевых показателей ОХ 3,3–5,2 ммоль/л, ЛПВП 1,15–2,60 ммоль/л, ЛПНП 1,1–3,0 ммоль/л, триглицеридов 0,1–1,7 ммоль/л). Всем пациентам проведена ультразвуковая доплерография брахиоцефальных артерий для оценки наличия/отсутствия атеросклеротического поражения сосудов.

Снижение почечной функции оценивалась путем расчета скорости клубочковой фильтрации (СКФ). Референсный интервал: 90–130 мл/мин/1,73 м².

Данные изображений КТ

Для первой части работы — анализа взаимосвязей между КТ-характеристиками и клинико-лабораторными данными, рассматривали характеристики изображений КТ:

## Статистический анализ

Статистический анализ данных проводили с использованием языка программирования Python 3.9.21. Описательная статистика категориальных данных представлена абсолютными и относительными частотами в виде n (%), а непрерывные переменные представлены медианами, первым и третьим квартилями в виде Me [ Q1; Q3].

Анализ двух независимых групп для количественных данных (в случае бинарного категориального признака) проводился с помощью критерия Манна-Уитни (U-тест), при сравнении трех независимых групп — тест Краскела-Уоллиса (H-тест). Для post-hoc теста использовался критерий Данна. Частоты бинарных признаков сравнивались между собой с помощью критерия χ². Корреляционный анализ количественных параметров выполнялся с помощью метода Спирмена. Применяли глобальную коррекцию на множественные сравнения методом Бенджамини-Хохберга. Пороговые значения (cut-off), соответствующие максимальному значению индекса Юдена, определяли по результатам ROC-анализа. 95% доверительные интервалы (ДИ) рассчитывали методом непараметрического бутстрапа с количеством выборок 1000.

Критический уровень статистической значимости при проверке статистических гипотез выбрали равным 0,05. Поправка на множественные сравнения применялась путем коррекции уровня значимости.

## Этическая экспертиза

Исследование рассмотрено и одобрено Локальным этическим комитетом ФГБУ «НМИЦ эндокринологии им. академика И.И. Дедова» Минздрава России (код протокола 20, 13.11.2024). Все пациенты дали письменное согласие на использование результатов обследования и лечения с научной целью.

## Результаты

Среди 254 пациентов, прооперированных ГНЦ ФГБУ «НМИЦ эндокринологии» Минздрава России, гормональная активность установлена на дооперационном этапе у 89,0% (n=226). ПГА установлен у 111 исследуемых, манифестный СК и ФАПК у 80 и 27 соответственно. Смешанная секреция ПГА+ФАПК диагностирована в 8 случаях. Характеристика пациентов (категориальных и непрерывных признаков) представлена в таблицах 1 и 2.

**Table:** Таблица 1. Характеристика пациентов (категориальные признаки) с адренокортикальными аденомами (n=254) Таблица 2. Характеристика пациентов (непрерывные признаки) с адренокортикальными аденомами (n=254) АКВ — абсолютный коэффициент вымывания; АКТГ — адренокортикотропный гормон; ОКВ — относительный коэффициент вымывания; ПДТ1 — подавляющий дексаметазоновый тест с 1 мг дексаметазона, ПроцАртНакопл — процент накопления контрастного вещества в артериальную фазу исследования; ПроцВенНакопл — процент накопления контрастного вещества в венозную фазу исследования

Признак	Описание (n, %)
Пол	Мужчина — 64 (25,2%), женщина — 190 (74,8%)
Тип секреции	• ПГА — 111 (43,7%), • Манифестный СК — 80 (31,5%), • ФАПК — 27 (10,6%), • Смешанный (ПГА+ФАПК) — 8 (3,1%), • Отсутствует — 28 (11,1%)
Наличие АГ	Да — 237 (93,3%), нет — 17 (6,7%)
Наличие нарушений углеводного обмена	Да — 75 (29,5%), нет — 179 (70,5%)
Наличие дислипидемии	Да — 168 (66,1%), нет — 86 (33,9%)
Наличие гипокалиемии в анамнезе	Да — 102 (40,2%), нет — 152 (59,8%)
Наличие кальцинатов	Да — 34 (13,4%), нет — 220 (85,6%)

Признак	Медиана	Межквартильный интервал [ Q1; Q3]
Клинические данные
Возраст, лет	48,7	[ 39,9; 58,9]
ИМТ	29,6	[ 25,6; 33,5]
СКФ	97,0	[ 78,5; 100,0]
Гормональное обследование
АКТГ (08:00), пг/мл	4,0	[ 1,1; 17,0]
Кортизол в ходе ПДТ1, пмоль/л	142,0	[ 47,8; 506,0]
Кортизол в суточной моче, пмоль/сут	473,5	[ 215,8; 1044,0]
Альдостерон, нмоль/л	586,5	[ 146,2; 462,2]
Прямой ренин, нмоль	2,5	[ 0,9; 8,2]
Калий, нмоль/л	3,8	[ 3,1; 4,3]
ПГА (n=111)
Альдостерон, нмоль/л	1246,0	[ 839,0; 2027,5]
Прямой ренин, нмоль	1,1	[ 0,5; 2,3]
Калий, нмоль/л	3,1	[ 2,8; 3,6]
Манифестный СК (n=80)
АКТГ (08:00) пг/мл	1,0	[ 1,0; 2,0]
Кортизол в ходе ПДТ1, пмоль/л	554,0	[ 439,3; 704,5]
Кортизол в суточной моче, пмоль/сут	952,0	[ 527,8; 1623,0]
Калий, нмоль/л	4,2	[ 3,9; 4,6]
ФАПК (n=27)
АКТГ (08:00), пг/мл	3,3	[ 2,1; 5,2]
Кортизол в ходе ПДТ1, пмоль/л	211,0	[ 139,0; 337,5]
Кортизол в суточной моче, пмоль/сут	429,0	[ 254,0; 584,5]
Калий, нмоль/л	4,4	[ 4,0; 4,5]
Смешанная секреция (n=8)
АКТГ (08:00) пг/мл	4,3	[ 2,2; 5,9]
Кортизол в ходе ПДТ1, пмоль/л	343,5	[ 108,3; 624,3]
Кортизол в суточной моче, пмоль/сут	424,0	[ 299,8; 565,0]
Альдостерон, нмоль/л	976,5	[ 243,5; 1645,0]
Прямой ренин, нмоль	1,2	[ 0,8; 1,3]
Калий, нмоль/л	4,0	[ 2,8; 4,3]
Отсутствие гормональной гиперсекреции (n=28)
АКТГ (08:00) пг/мл	20,8	[ 10,3; 38,1]
Кортизол в ходе ПДТ1, пмоль/л	58,0	[ 33,0; 98,0]
Кортизол в суточной моче, пмоль/сут	212,0	[ 182,5; 390,0]
Альдостерон, нмоль/л	193,0	[ 141,5; 316,5]
Прямой ренин, нмоль	10,5	[ 5,1; 80,7]
Калий, нмоль/л	4,2	[ 4,1; 4,4]
Характеристики КТ-признаков
Mаксимальный размер, мм	29,5	[ 20,0; 37,8]
Минимальная нативная плотность, HU	-7,0	[ -18,0; 4,0]
Максимальная нативная плотность, HU	29,0	[ 14,0; 40,0]
Максимальная плотность в артериальную фазу, HU	72,0	[ 50,0; 100,0]
Максимальная плотность в венозную фазу, HU	98,0	[ 71,8; 122,5]
Максимальная плотность в отсроченную фазу, HU	56,0	[ 40,0; 74,0]
ПроцАртНакоп, %	161,4	[ 65,5; 333,4]
ПроцВенНакоп, %	255,8	[ 146,2; 462,9]
ОКВ, %	45,0	[ 32,2; 55,6]

Первым этапом проведен сравнительный анализ с помощью критерия Краскела-Уоллиса (H-тест) непрерывных и категориальных характеристик среди двух групп пациентов — с и без гормональной активности. Получены статистически значимые результаты для следующих пар признаков (табл. 3).

**Table:** Таблица 3. Сравнительный анализ пациентов с аденомами надпочечников в зависимости от наличия/отсутствия гормональной активности

Характеристика	Гормональная активность (n,%)	Sensitivity	Specificity	PPV	NPV	Cut-off	р
Да (n=226, 89,0%)	Нет (n=28, 11,0%)
Максимальный размер	29,0 [ 20,0; 36,0]	43,0 [ 32,7; 51,2]	0,616 (0,552; 0,680)	0,792 (0,632; 0,938)	0,964 (0,933; 0,992)	0,184 (0,116; 0,263)	31,0	<0,001
MAX плотность в нативную фазу	25,0 [ 12,0; 37,0]	41,0 [ 36,0; 47,2]	0,724 (0,665; 0,783)	0,786 (0,621; 0,931)	0,964 (0,933; 0,988)	0,265 (0,172; 0,349)	35,0	<0,001
МАХ плотность в отсроченную фазу	55,0 [ 35,0; 72,7]	67,0 [ 52,0; 80,5]	0,423 (0,352; 0,492)	0,926 (0,812; 1,000)	0,976 (0,937; 1,000)	0,182 (0,122; 0,252)	48,0	0,022
ПроцАртНакоп, %	182,2 [ 76,1; 353,2]	93,4 [ 31,6; 156,8]	0,426 (0,356; 0,497)	0,923 (0,800; 1,000)	0,976 (0,938; 1,000)	0,182 (0,114; 0,248)	214,8	0,013
ПроцВенНакоп, %	283,7 [ 163,4; 480,3]	141,6 [ 113,9; 250,7]	0,668 (0,599; 0,732)	0,731 (0,545; 0,893)	0,948 (0,906; 0,979)	0,232 (0,139; 0,322)	196,7	0,001

При сравнительном анализе выявлено, что гормонально неактивные аденомы были значительно больше, имели высокую плотность в нативной и отсроченной фазах, при этом имели меньший % накопления контрастного препарата в венозную и артериальную фазу (боксплоты представлены на рисунке 1).

**Figure:**
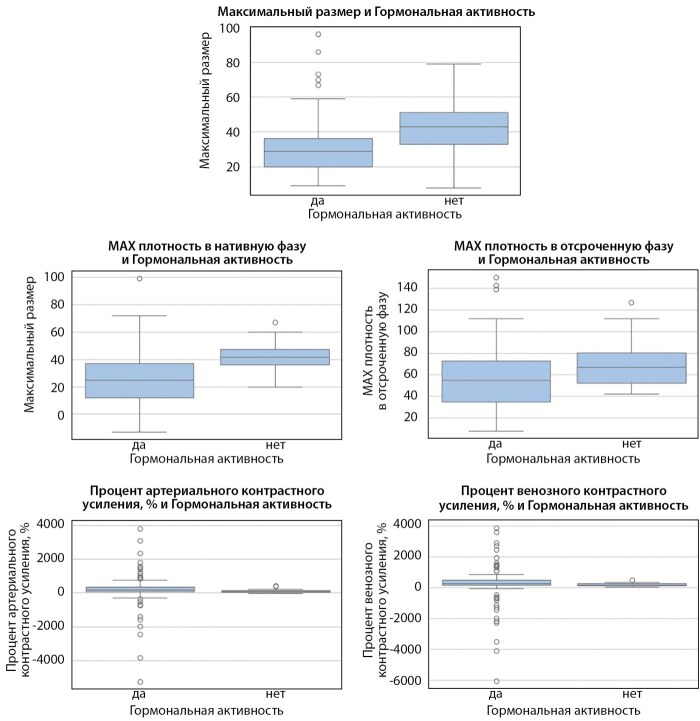
Рисунок 1. Боксплоты к таблице 3.

Вторым этапом анализа аденомы были разделены на соответствующие пять групп в зависимости от гормональной активности. Проведен сравнительный анализ КТ-признаков с помощью критерия Краскела-Уоллиса (H-тест) всех непрерывных КТ-характеристик среди 5 групп пациентов: с ПГА, манифестным СК, ФАПК, смешанной гормональной секрецией и без гормональной активности (табл. 4).

**Table:** Таблица 4. Сравнительный анализ КТ-характеристик аденом надпочечников с различной гормональной активностью ¹ критерий Краскелла-Уоллиса ² критерий χ²

Признак	Me [ Q1; Q3]/n (%)	р	p, post-hoc
ПГА (группа 1) (N=111)	ФАПК + ПГА (группа 2) (N=8)	ФАПК (группа 3) (N=27)	Манифестный СК (группа 4) (N=80)	Без горм активности (группа 5) (N=28)
Максимальный размер	20,0 [ 16,8; 25,0]	38,0 [ 29,5; 41,5]	42,0 [ 30,0; 47,5]	34,0 [ 30,0; 38,0]	43,0 [ 32,7; 51,2]	<0,001¹	p1-2<0,001 p1-3<0,001 p1-4<0,001 p1-5<0,001 p2-3=0,923 p2-4=0,830 p2-5=0,923 p3-4=0,331 p3-5=0,939 p4-5=0,331
MAX плотность в нативную фазу	14,0 [ 4,0; 24,0]	27,0 [ 17,7; 40,5]	29,5 [ 14,5; 40,0]	35,5 [ 30,0; 44,0]	41,0 [ 36,0; 47,2]	<0,001¹	p1-2=0,064 p1-3=0,001 p1-4<0,001 p1-5<0,001 p2-3=0,991 p2-4=0,260 p2-5=0,081 p3-4=0,081 p3-5=0,013 p4-5=0,179
MAX плотность в венозную фазу	84,0 [ 61,0; 104,5]	85,0 [ 75,0; 120,5]	85,0 [ 57,7; 118,0]	117,0 [ 100,0; 146,0]	102,0 [ 94,0; 122,5]	<0,001¹	p1-2=0,690 p1-3=0,731 p1-4<0,001 p1-5=0,015 p2-3=0,731 p2-4=0,165 p2-5=0,513 p3-4=0,003 p3-5=0,153 p4-5=0,312
MIN плотность в нативную фазу	-4,0 [ -10; 6]	-8,0 [ -19,5; 7,75]	-28,50 [ -49,7; -8,2]	-10,5 [ -21,2; 1,0]	-14,5 [ -39,5; 2,6]	<0,001¹	p1-2=0,477 p1-3<0,001 p1-4=0,009 p1-5=0,009 p2-3=0,071 p2-4=0,699 p2-5=0,477 p3-4=0,009 p3-5=0,106 p4-5=0,477
MAX плотность в отсроченную фазу	44,5 [ 29,7; 65,5]	69,0 [ 30,5; 71,5]	55,5 [ 40,0; 75,0]	65,0 [ 50,0; 78,0]	67,0 [ 52,0; 80,5]	<0,001¹	p1-2=0,577 p1-3=0,273 p1-4<0,001 p1-5=0,001 p2-3=0,823 p2-4=0,424 p2-5=0,326 p3-4=0,326 p3-5=0,273 p4-5=0,577
ПроцАртНакоп, %	275,6 [ 148,7; 428,6]	92,8 [ 26,0; 440,6]	115,9 [ 38,6; 322,3]	122,2 [ 65,7; 202,3]	93,4 [ 31,6; 156,8]	<0,001¹	p1-2=0,353 p1-3=0,090 p1-4=0,001 p1-5=0,001 p2-3=0,990 p2-4=0,931 p2-5=0,553 p3-4=0,905 p3-5=0,353 p4-5=0,353
ПроцВенНакоп, %	382,5 [ 214,6; 604,1]	166,0 [ 91,0; 391,1]	255,8 [ 109,9; 602,0]	226,4 [ 159,4; 361,2]	141,6 [ 113,9; 250,7]	0,001¹	p1-2=0,219 p1-3=0,231 p1-4=0,025 p1-5<0,001 p2-3=0,657 p2-4=0,699 p2-5=0,606 p3-4=0,699 p3-5=0,084 p4-5=0,078
АКВ, %	54,69 [ 44,3; 66,4]	60,7 [ 38,1; 80,2]	62,5 [ 26,9; 75,4]	64,6 [ 53,8; 77,3]	64,8 [ 48,3; 77,5]	0,032¹	p1-2=0,724 p1-3=0,780 p1-4=0,016 p1-5=0,488 p2-3=0,778 p2-4=0,778 p2-5=0,979 p3-4=0,468 p3-5=0,724 p4-5=0,724
Кальцинаты	5 (4,5%)	2 (25%)	7 (25,9%)	12 (15,0%)	7 (25,0%)	0,009²	p1-2=1,000 p1-3=0,010 p1-4=0,223 p1-5=0,031 p2-3=1,000 p2-4=1,000 p2-5=1,000 p3-4=1,000 p3-5=1,000 p4-5=1,000

Статистически значимые различия выявлены по размеру образования, минимальной и максимальной плотности в нативную фазу, максимальной плотности в венозную и отсроченную фазы исследований. Также группы имели различия по проценту артериального и венозного накопления. Таким образом, при синдроме Конна аденомы надпочечника были значительно меньше по размеру, а также нативная плотность была ниже, чем при других гормонально-активных образованиях коркового слоя надпочечника. Аденомы, происходящие из пучкового слоя надпочечников, имеют большую плотность в нативной, венозной (p<0,001) и отсроченной (р=0,001) фазах, также значительно больше накапливают контрастный препарат в артериальную и венозную фазу по сравнению с опухолями из клубочкового слоя.

Наличие кальцинатов в образованиях различалось на уровне статистической достоверности (p=0,009). Для ПГА характерно пониженная частота встречаемости кальцинатов по сравнению с ФАПК и отсутствием гормональной секреции.

С учетом наличия статистически значимых корреляций между видом гормональной активности и размером образования, а также наличия отличий по частоте встречаемости кальцинатов, нами был проведен корреляционный анализ данных показателей (табл. 5).

**Table:** Таблица 5. Анализ между размером образования и наличием кальцинатов (тест Манна-Уитни p-value <0,001)

Характеристика	Наличие кальцинатов
да	нет
Максимальный размер, мм	36,0 (29,0–46,0)	29,0 (20,0–36,5)

Таким образом, увеличение вероятности наличия кальцинатов связано с увеличением размеров образования (рис. 2).

**Figure:**
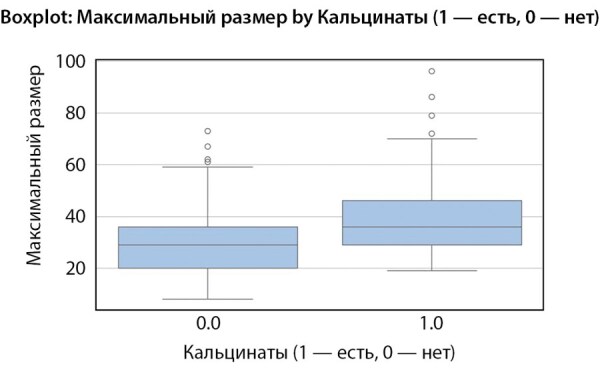
Рисунок 2. Боксплот: связь между размером образования и наличием кальцинатов.

Далее нами было проведено исследование аденом с различными типами секреции по анализируемым признакам изображений КТ с применением теста Манна-Уитни (U-тест), результаты приведены в таблице ниже (табл. 6).

**Table:** Таблица 6. Результаты статистически значимых результатов и тенденций сравнительного анализа (тест Манна-Уитни) пациентов с различными коморбидными состояниями по анализируемым признакам изображений КТ

Характеристика	Значение категориального признака	Чувств-ть	Спец-ть	PPV	NPV	Cut-off	р
да	нет
Me [ Q1; Q3] Me [ Q1; Q3]	Me [ Q1; Q3] Me [ Q1; Q3]
Гипокалиемия в анамнезе (да — 102, нет — 152)
Максимальный размер	21,0 [ 17,0; 28,2]	34,0 [ 28,0; 42,0]	0,700 (0,613; 0,784)	0,797 (0,729; 0,859)	0,707 (0,615; 0,791)	0,792 (0,728; 0,853)	26	<0,001
MIN плотность в нативную фазу	-4,0 [ -10; 6,0]	-11,0 [ -27,5; 0,5]	0,792 (0,708; 0,869)	0,490 (0,408; 0,566)	0,516 (0,438; 0,594)	0,774 (0,680; 0,857)	-11	0,001
MAX плотность в нативную фазу	15,0 [ 5,0; 25,0]	36,0 [ 24,5; 44,5]	0,851 (0,780; 0,918)	0,653 (0,579; 0,726)	0,628 (0,548; 0,709)	0,865 (0,798; 0,927)	30	<0,001
MAX плотность в венозную фазу	84,0 [ 62,0; 106,0]	109,0 [ 87; 133,7]	0,652 (0,553; 0,747)	0,715 (0,639; 0,790)	0,611 (0,515; 0,710)	0,750 (0,675; 0,821)	92	<0,001
MAX плотность в отсроченную фазу	44,5 [ 30,0; 61,5]	65,0 [ 49,0; 78,7]	0,589 (0,494; 0,681)	0,762 (0,687; 0,831)	0,631 (0,533; 0,728)	0,728 (0,652; 0,805)	48	<0,001
Процент артериального контрастного усиления, %	289,1 [ 151,2; 430,7]	115,8 [ 53,4; 210,2]	0,776 (0,671; 0,859)	0,602 (0,516; 0,686)	0,564 (0,474; 0,658)	0,802 (0,717; 0,874)	148,3	<0,001
Процент венозного контрастного усиления, %	381,8 [ 228,2; 535]	207,2 [ 131,2; 337,8]	0,598 (0,500; 0,694)	0,758 (0,677; 0,833)	0,627 (0,521; 0,732)	0,735 (0,659; 0,803)	340,9	<0,001
Нарушение углеводного обмена (да — 75, нет — 179)
Максимальный размер	34,0 [ 28,5; 42,5]	27,0 [ 19,0; 35,0]	0,775 (0,680; 0,870)	0,529 (0,456; 0,602)	0,404 (0,322; 0,491)	0,850 (0,784; 0,917)	28	<0,001
MAX плотность в нативную фазу	32,0 [ 20,0; 44,0]	24,0 [ 10,7; 37,0]	0,644 (0,537; 0,754)	0,580 (0,511; 0,651)	0,388 (0,294; 0,476)	0,797 (0,731; 0,865)	30	0,003
MAX плотность в венозную фазу	112,0 [ 85,0; 132,2]	93,5 [ 68,0; 118,7]	0,576 (0,457; 0,698)	0,669 (0,592; 0,743)	0,427 (0,326; 0,531)	0,786 (0,710; 0,856)	109	0,015
MAX плотность в отсроченную фазу	66,5 [ 53,2; 79,7]	50,0 [ 34,0; 71,5]	0,773 (0,671; 0,870)	0,535 (0,455; 0,614)	0,415 (0,326; 0,500)	0,847 (0,770; 0,913)	52	0,002
Артериальная гипертензия (да — 237, нет — 17)
MAX плотность в нативную фазу	27,0 (12,8–38,3)	40,5 (36,0–44,3)	0,908 (0,871; 0,944)	0,308 (0,067; 0,583)	0,959 (0,931; 0,986)	0,160 (0,032; 0,310)	46	0,001
Процент венозного контрастного усиления, %	275,6 [ 153,9; 478,1]	144,3 [ 117; 199,7]	0,608 (0,539; 0,682)	0,812 (0,615; 1,000)	0,976 (0,947; 1,000)	0,143 (0,076; 0,215)	226,5	0,011
Процент артериального контрастного усиления, %	173,5 [ 76,6; 352,6]	85,0 [ 28,9; 153,2]	0,360 (0,296; 0,427)	1,000 (1,000; 1,000)	1,000 (1,000; 1,000)	0,113 (0,060; 0,168)	260	0,024
Дислипидемия (да — 168, нет — 86)
MAX плотность в отсроченную фазу	60,0 [ 44,0; 78,0]	46,5 [ 32,7; 66,2]	0,703 (0,636; 0,774)	0,539 (0,421; 0,653)	0,745 (0,667; 0,818)	0,488 (0,379; 0,596)	49	0,006
MAX плотность в венозную фазу	101,5 [ 80,0; 133,0]	91 [ 59,7; 112,2]	0,403 (0,322; 0,483)	0,776 (0,684; 0,865)	0,773 (0,676; 0,865)	0,407 (0,320; 0,487)	114	0,020

При сравнительном анализе выявлено, что гипокалиемия чаще наблюдалась среди образований маленького размера и малой нативной плотности, что в целом коррелирует с данными, полученными ранее (табл. 4), где данные признаки были связаны с ПГА. В то время, как нарушение углеводного обмена чаще наблюдалось при образованиях большего размера (p<0,001).

Следующим этапом проведен анализ пар непрерывных признаков (табл. 7).

**Table:** Таблица 7. Результаты теста Спирмена

КТ-признак	Лабораторные показатели	R, коэффициент корреляции	p-value
Максимальный размер	Альдостерон (пмоль/л)	-0,504	<0,001
MAX плотность в нативную фазу	Альдостерон (пмоль/л)	-0,457	<0,001
Максимальный размер	АКТГ (пг/мл)	-0,419	<0,001
MAX плотность в нативную фазу	АКТГ (пг/мл)	-0,387	<0,001
MAX плотность в венозную фазу	АКТГ (пг/мл)	-0,304	<0,001
Процент артериального контрастного усиления, %	Ренин (нмоль/л)	-0,293	<0,001
MAX плотность в отсроченную фазу	АКТГ (пг/мл)	-0,268	0,001
Процент артериального контрастного усиления, %	Калий (нмоль/л)	-0,268	0,001
MAX плотность в венозную фазу	Альдостерон (пмоль/л)	-0,259	0,001
MAX плотность в отсроченную фазу	Альдостерон (пмоль/л)	-0,258	0,001
Процент венозного контрастного усиления, %	Ренин (нмоль/л)	-0,248	0,002
Процент венозного контрастного усиления, %	Калий (нмоль/л)	-0,248	0,001
MIN плотность в нативную фазу	Калий (нмоль/л)	-0,239	0,001
Процент артериального контрастного усиления, %	СКФ (мл/мин/1,73 м²)	-0,188	0,020
MAX плотность в нативную фазу	ИМТ (кг/ м²)	0,146	0,049
Максимальный размер	ИМТ (кг/ м²)	0,166	0,024
Абсолютный коэффициент вымывания, %	Кортизол в ходе ПДТ1 (нполь/л)	0,187	0,024
Абсолютный коэффициент вымывания, %	СКФ (мл/мин/1,73 м²)	0,193	0,015
Процент венозного контрастного усиления, %	АКТГ (ПГ/МЛ)	0,197	0,024
MAX плотность в венозную фазу	Кортизол в суточной моче (нмоль/сут)	0,216	0,042
MAX плотность в артериальную фазу	Кортизол в ходе ПДТ1 (нполь/л)	0,224	0,006
MAX плотность в венозную фазу	ИМТ (кг/м²)	0,229	0,002
MAX плотность в отсроченную фазу	Ренин (нмоль/л)	0,245	0,002
MAX плотность в артериальную фазу	Кортизол в суточной моче (нмоль/сут)	0,248	0,017
Процент венозного контрастного усиления, %	Альдостерон (пмоль/л)	0,248	0,002
MAX плотность в венозную фазу	Калий (нмоль/л)	0,269	<0,001
MAX плотность в венозную фазу	Ренин (нмоль/л)	0,274	0,001
MAX плотность в отсроченную фазу	Кортизол в суточной моче (нмоль/сут)	0,275	0,007
Процент артериального контрастного усиления, %	АКТГ (ПГ/МЛ)	0,277	0,001
MAX плотность в отсроченную фазу	Кортизол в ходе ПДТ1 (нполь/л)	0,290	<0,001
MIN плотность в нативную фазу	Альдостерон (пмоль/л)	0,294	<0,001
MAX плотность в отсроченную фазу	Калий (нмоль/л)	0,295	<0,001
Процент артериального контрастного усиления, %	Альдостерон (пмоль/л)	0,305	<0,001
MAX плотность в нативную фазу	Кортизол в суточной моче (нмоль/сут)	0,341	<0,001
MAX плотность в венозную фазу	Кортизол в ходе ПДТ1 (нполь/л)	0,382	<0,001
MAX плотность в нативную фазу	Кортизол в ходе ПДТ1 (нполь/л)	0,433	<0,001
Максимальный размер	Ренин (нмоль/л)	0,454	<0,001
Максимальный размер	Калий (нмоль/л)	0,458	<0,001
MAX плотность в нативную фазу	Калий (нмоль/л)	0,459	<0,001
MAX плотность в нативную фазу	Ренин (нмоль/л)	0,459	<0,001
Максимальный размер	Кортизол в ходе ПДТ1 (нполь/л)	0,500	<0,001

В результате вычисления коэффициентов корреляции Спирмена наиболее сильная отрицательная связь выявлена между максимальным размером образования и максимальной плотностью в нативной фазе исследования и уровнями секреции альдостерона и АКТГ. В свою очередь максимальные значения коэффициента корреляции по модулю характерны для положительной связи максимального размера и уровнями секреции кортизола в ходе ПДТ1, ренина, калия; между максимальной плотностью в нативную фазу и уровнями секреции калия, ренина (коэффициент корреляции 0,459 при p<0,001). Диаграммы рассеяния для пар признак с наиболее сильной корреляцией приведены на рисунке 3.

**Figure:**
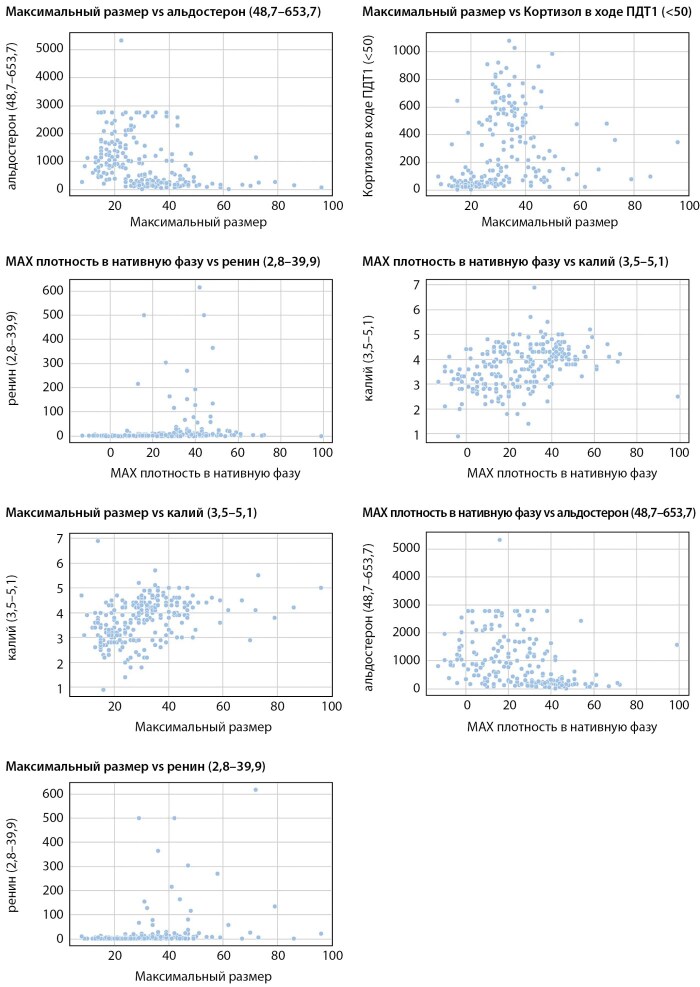
Рисунок 3. Диаграммы корреляционного анализа максимального размера и максимальной плотностью в нативную фазу и уровнями секреции альдостерона, ренина, кортизола в ходе ПДТ1 и АКТГ и калия у пациентов с гормонально-активными аденомами надпочечников.

## Обсуждение

## Репрезентативность выборок

Аденомы надпочечников составляют примерно от 54 до 75% инциденталом надпочечников, среди которых нечасто наблюдается гиперсекреция гормонов. Преобладание гормонально-активных аденом в нашей работе (89%) обусловлено в первую очередь госпитальным характером выборки пациентов. В данное ретроспективное исследование были включены только прооперированные пациенты с верифицированным морфологическим диагнозом «аденома надпочечника», что резко сокращает в выборке количество пациентов с образованиями без гормональной активности. В популяционных исследованиях процент гормонально-активных форм, как правило, колеблется около 15–25% [4][5]. В связи с чем, учитывая ограниченную репрезентативность выборки, полученные результаты невозможно экстраполировать на общую популяцию.

ПГА встречается по крайней мере у 10% всех пациентов с гипертонией, хотя статистические данные о оценках распространенности сильно различаются. Частота ПГА увеличивается с тяжестью сопутствующей гипертонии. Распространенность превышает 20% у пациентов с медикаментозно резистентной гипертонией и может достигать 50%, особенно у лиц в возрасте <40 лет или у пациентов с гипокалиемией [6]. По данным исследования Rossi и соавт., среди впервые диагностированных пациентов с гипертонией ПГА обнаруживается примерно у 11 200 на 100 000 человек [7], что противоречит ранее проведенным исследованиям, где данный показатель не превышал 300 на 100 000 [8]. В свою очередь, по данным популяционных исследований, общая частота эндогенного СК составляет приблизительно 1,8–3,2 случая на 1 000 000 человек в год, а распространенность — около 57–79 случаев на миллион (≈5,7–7,9 на 100 000) уровня общей популяции [9]. Исследование в Швеции показало ежегодную частоту примерно 0,7 случая на миллион человек в год, обусловленных кортикостеромой надпочечника [5]. Андроген продуцирующие аденомы встречаются редко (менее 1%).

Среди 254 прооперированных пациентов, включенных в нашу выборку, диагноз «ПГА» установлен у 111 человек (43,7%), «СК» — у 107, среди которых манифестная форма выявлена у 80 (31,5%), а «ФАПК» (субклиническая форма СК) — у 27 (10,6%). Опухоли смешанной секреции составили 3,1% (8/254). Такое распределение в выборке обусловлено тем, что пациенты с ФАПК получали хирургическое лечение только в случае наличия тяжелого течения коморбидных заболеваний (АГ, СД, ожирение, минерально-костные-нарушения), или наличия супрессированого уровня АКТГ, что в данной группе пациентов бывает в 25%.

Доля ГНА составила 11,2% и была представлена образованиями с неопределенным КТ-фенотипом (опухоли высокой плотности менее 4 см; опухоли низкой плотности более 4 см; опухоли мозаичной плотности (вследствие неоднородной структуры)). ГНА с низким содержанием жира представляют значительные трудности в дифференциальной диагностике с адренокортикальным раком (АКР). Визуально такие образования могут иметь сходные размеры, форму и структуру с АКР, особенно на ранних стадиях.

Cредний возраст исследуемых пациентов составил 48,7 года [ 39,9; 58,9], с преобладанием среди исследуемых женщин (соотношение 3 к 1), что соответствует данным большинства ранее опубликованных исследований, хотя отмечено несколько работ, где преобладали пациенты мужского пола, особенно в крупном корейском исследовании Song и соавт. [10–13].

## Сопоставление с другими публикациями

При сравнительном анализе клинико-радиологических характеристик между двумя группами пациентов — с гормонально-активными и ГНА надпочечников — выявлены статистически значимые различия (p<0,001): аденомы в группе без гормональной активности характеризовались большими размерами (43 мм [ 32,7; 51,2] против 29 мм [ 20; 36] соответственно), более высокой нативной плотностью по данным КТ (41 HU [ 36; 47,2] против 25 HU [ 12; 37]) , а также меньшим процентом накопления контрастного вещества в венозную фазу по сравнению с гормонально-активными образованиями, что в целом соответствует данным ранее опубликованных исследований [14]. Так, в исследовании Chen et al. (2022) было показало, что плотность на нативных КТ-изображениях была значимо выше у ГНА (ГНА: медиана ≈ 5,19 HU & альдостеромы: ≈ 1,41 HU, p<0,05), при этом альдостеромы были меньше по размеру [15].

По результатам нашего исследования, при синдроме Конна аденомы значительно меньше по размеру и имеют более низкую нативную плотность, чем при кортикостеромах. Кортикостеромы демонстрируют значительно более высокие значения плотности в нативной и венозной фазе (p<0,001), отсроченной фазе (p=0,001), а также существенно большее артериальное накопление контрастного вещества по сравнению с альдостеромами. При анализе зарубежной литературы, в исследовании Li и соавт., при сравнении КТ-характеристик вышеуказанных гормонально-активных аденом, получены аналогичные данные [16]. Разница в значениях характеристик КТ связана с гистоцитологическими различиями — альдостеромы богаты прозрачными клетками с большим объемом и равномерным распределением липидных компонентов, тогда как кортикостеромы состоят из зернистых клеток с небольшим объемом и дисперсным распределением липидных компонентов [14][17].

В результате корреляционного анализа установлены статистически значимые умеренные положительные связи между размером аденомы, максимальной плотностью в нативной фазе исследования и уровнем кортизола в ходе ПДТ1 (r=0,50 и 0,46, p<0,001), а также умеренно отрицательная связь между размерами узловой ткани и уровнем АКТГ (r=–0,41 и -0,38 p<0,001), что подтверждает данные исследования Olmos и соавт., где также была отмечена положительная корреляция между объемом аденомы и уровнем кортизола после теста с дексаметазоном, а также отрицательная корреляция с уровнем АКТГ (r≈0,46 и 0,28, p<0,001) [18]. Аналогичные наблюдения описаны в ряде других работ [19–21]. Эти результаты свидетельствуют о том, что увеличение объема узловой ткани способствует стимуляции стероидогенеза и подавлению секреции АКТГ, что может усугублять клиническое течение гиперкортицизма.

При анализе альдостерон-продуцирующих надпочечниковых образований выявлены статистически значимые отрицательные корреляции между размерами образования и его максимальной нативной плотностью с уровнем альдостерона в крови (r=–0,50 и –0,45 соответственно; p<0,001). Наряду с этим установлены положительные корреляции между вышеуказанными морфологическими характеристиками и концентрацией ренина, а также уровнем калия в сыворотке крови (r=0,45; p<0,001). Представленные данные согласуются с международными исследованиями последних лет. Обратные корреляции между объемом/плотностью альдостерон-продуцирующих узлов и уровнем альдостерона, а также прямые — с ренином и калием, вероятно, отражают биологическую неоднородность этих образований [22][24].

## Клиническая значимость результатов

Полученные результаты позволяют на этапе лучевой диагностики сформировать более точное представление о вероятном типе гормональной активности опухоли, что имеет прямое влияние на выбор дальнейшей диагностической и лечебной тактики. Установленные закономерности, такие как малый размер и низкая нативная плотность, характерные для альдостерон-продуцирующих аденом, или, напротив, большие размеры и высокая плотность, ассоциированные с кортизол-продуцирующими опухолями и гормонально-неактивными образованиями, предоставляют ценные диагностические ориентиры. Выявленные корреляции, в частности, отрицательная связь между размером аденомы/ее плотностью и уровнем альдостерона, а также положительная связь с уровнем кортизола, ренина и калия, углубляют понимание патофизиологических взаимосвязей между морфологией опухоли и ее функциональным статусом. Таким образом, интеграция количественных КТ-параметров в алгоритм обследования позволяет персонализировать подход к каждому пациенту.

## Ограничения исследования

Данное исследование, несмотря на полученные значимые результаты, имеет ряд ограничений, которые необходимо учитывать при интерпретации его выводов. Основным ограничением является госпитальный характер выборки, состоящей исключительно из пациентов, направленных на хирургическое лечение в специализированный эндокринологический центр. Это привело к значительному преобладанию гормонально-активных аденом (89%) над гормонально-неактивными (11%), что не отражает реальную распространенность данных образований в общей популяции, где, напротив, доминируют нефункционирующие инциденталомы. Следовательно, результаты исследования не могут быть экстраполированы на общую популяцию пациентов со случайно обнаруженными образованиями надпочечников, и их применимость в первую очередь касается пациентов с установленными или высоковероятными гормонально-активными синдромами. Ретроспективный дизайн исследования не исключает риска систематических ошибок, связанных с отбором пациентов. Использование различных моделей КТ-сканеров, хотя и является обычной практикой, потенциально могло внести некоторую вариабельность в измерения плотности. Объем выборки, особенно для подгрупп с редкими типами секреции, является относительно небольшим, что могло ограничить статистическую мощность для выявления значимых различий в этих категориях. Кроме того, в работе не оценивалась согласованность при оценке КТ-параметров что могло привести к случайным ошибкам измерения.

## Направления дальнейших исследований

Остаются актуальными нерешенные задачи, связанные с оптимизацией диагностики и разработкой персонализированных подходов к ведению пациентов с надпочечниковыми образованиями. Необходимы более углубленные исследования КТ-характеристик, морфологической и функциональной структуры опухолей коры надпочечников, а также выявление факторов, определяющих клиническое течение заболевания. Это позволит более точно формулировать тактику наблюдения и лечения, обоснованно выбирать индивидуальную терапию и своевременно корректировать ее с учетом прогностических маркеров и динамики состояния пациента.

## Заключение

В данной работе представлены результаты ретроспективного одноцентрового исследования, направленного на изучение клинико-лабораторных и инструментальных характеристик пациентов с аденомами надпочечников. Проведенный анализ выявил статистически значимые ассоциации между клинико-лабораторными и совокупностью показателей и параметров, полученных при КТ.

## Дополнительная информация

Источники финансирования. Работа выполнена по инициативе авторов без привлечения финансирования.

Конфликт интересов. Авторы декларируют отсутствие явных и потенциальных конфликтов интересов, связанных с содержанием настоящей статьи.

Участие авторов. Все авторы одобрили финальную версию статьи перед публикацией, выразили согласие нести ответственность за все аспекты работы, подразумевающую надлежащее изучение и решение вопросов, связанных с точностью или добросовестностью любой части работы.
